# ERO1L Promotes Hepatic Metastasis through Activating Epithelial-Mesenchymal Transition (EMT) in Pancreatic Cancer

**DOI:** 10.1155/2021/5553425

**Published:** 2021-02-23

**Authors:** JianYu Yang, Yu Xu, YanMiao Huo, Li Cai, Rong Hua, JunFeng Zhang, Zhong Chen

**Affiliations:** ^1^The First Affiliated Hospital of Soochow University, Suzhou, China; ^2^Department of Biliary-Pancreatic Surgery, Ren Ji Hospital, School of Medicine, Shanghai Jiao Tong University, Shanghai, China; ^3^Kong Jiang Hospital of Yangpu District, Shanghai, China; ^4^Department of General Surgery, Affiliated Hospital of Nantong University, Nantong, China

## Abstract

**Background:**

Endoplasmic reticulum oxidoreductase 1 alpha (ERO1L) serves as an effector for tumor growth in human malignancies. However, the mechanism of ERO1L on promoting metastasis of pancreatic ductal adenocarcinoma (PDAC) remains to be further explored.

**Methods:**

Bioinformatics analysis of public databases and large-scale metastatic PDAC sequencing was performed to determine the expression profile and prognostic value of ERO1L in PDAC. The effect of ERO1L on metastasis of PDAC was analyzed in vitro and in vivo, via cell biological, molecular, and biochemical approaches.

**Results:**

ERO1L in PDAC hepatic metastatic tissues were highly expressed and related to disease-free survival (DFS). Genetic silencing and pharmacological inhibition of ERO1L with EN460 suppressed cell migration and invasion of PDAC. Furthermore, EN460 also suppressed hepatic metastasis of PDAC in vivo. Using shRNAs and EN460 to inhibit the ERO1L expression in Capan-2 and MiaPaca-2 led to the remarkable change of EMT-related protein Vimentin and E-cadherin, which indicated that EMT acted as a key pathway for ERO1L to promote invasion, dissemination, colonization, and growth of hepatic metastasis in PDAC.

**Conclusion:**

Our findings uncover ERO1L contributes to hepatic metastasis in PDAC via epithelial-mesenchymal transition (EMT) process and indicate a promising therapeutic strategy for PDAC hepatic metastasis.

## 1. Introduction

Pancreatic ductal adenocarcinoma (PDAC) is one of the most lethal solid tumors. Distant metastasis is the leading cause of cancer death. Approximately 50% of pancreatic PDAC patients are discovered to have distant metastases, mainly in the liver [1]. Chemotherapy is the only recommended treatment for metastatic PDACs, with a median survival time of only 4–6 months and the 5-year survival of 1% [2]. Therefore, deciphering the molecular mechanisms of metastatic PDAC is urgently for exploring effective therapeutic targets.

Endoplasmic reticulum oxidoreductase 1 alpha (ERO1L), a flavin adenine nucleotide-containing enzyme, is located in the endoplasmic reticulum (ER)—an organelle entrusted with lipid synthesis, calcium storage, and appropriate processing of membrane and secreted proteins for maturation [3] and can be activated by ER stress under hypoxia, metabolic disorders, oxidative stress, or other pathological tumor microenvironment [4, 5]. ERO1L, together with protein disulfide isomerase (PDI), plays a role in disulfide bond formation of secreted and membrane proteins. ERO1L undergoes redox reaction, through receiving electrons from reduced PDI and transferring them on to molecular oxygen [6]. ERO1L is highly expressed in various types of human malignances, including but not limited to hepatocellular carcinoma (HCC), pancreatic cancer, gastric cancer, and breast cancer, which are associated with tumor progression and metastasis [7–10]. A recent research has demonstrated that oxidative protein folding of vascular endothelial growth factor (VEGF) and enhancement of VEGF messenger RNA (mRNA) expression can upregulate the expression of ERO1L, further promoting tumor growth [7]. Our previous study also demonstrated ERO1L-induced growth advantage is largely dependent on enhanced aerobic glycolysis in PDAC [10]. ERO1L also plays vital role in metastatic process. Another study has proved that in a well-established HCC xenograft model, the number of lung metastasis nodules is much higher in the ERO1L-overexpressing group, in comparation with the control group, which means ERO1L has the ability to promote HCC metastasis [11]. Therefore, ERO1L is a potential therapeutic target in future tumor treatment, but the molecular mechanism of ERO1L in distant metastasis in PDAC remains to be clarified and investigated.

Our previous study preliminarily explored the growth-promoting effect of ERO1L in PDAC. Our deep sequencing data for hepatic metastasis of PDAC suggested ERO1L may act a metastasis-promoting role in PDAC [12]. In this study, we further investigated the importance of ERO1L during PDAC metastasis. We conditionally inactivated ERO1L in PDAC models and discovered negative effect of ERO1L on PDAC hepatic metastasis. In addition, growing evidence verified ERO1L might promote metastasis through EMT.

## 2. Materials and Methods

### 2.1. Clinical Specimens

Primary and hepatic metastatic PDAC tissues and corresponding adjacent nontumor tissues were obtained from patients who underwent synchronous surgical resection of hepatic oligometastatic PDAC between January 2016 and December 2017 in Shanghai Cancer Institute, Ren Ji Hospital, School of Medicine, Shanghai Jiao Tong University, with informed consent. All patients involved had not received radiotherapy, chemotherapy, or other related antitumor therapies before surgery. All data were collected prospectively in a database that was approved by the Institutional Review Board.

### 2.2. Cell Lines and Cultures

Human pancreatic cancer cell lines Capan-2 and MiaPaCa-2 were all preserved in Shanghai Cancer Institute, Ren Ji Hospital, School of Medicine, Shanghai Jiao Tong University. Cells were cultured in suggested medium according to American Type Culture Collection (ATCC) protocols, supplemented with 10% (*v*/*v*) fetal bovine serum (FBS) and 1% (*v*/*v*) streptomycin-penicillin (Sigma-Aldrich, Shanghai, China) at 37°C in a humidified incubator under 5% CO2 condition.

### 2.3. Lentiviral Transfection-Mediated Knockdown

Short hairpin RNA (shRNA) against ERO1L gene or control vectors were transfected along with a three-plasmid system (pPACKH1-GAG, pPACKH1-REV, and pVSV-G) into HEK293T cells using Lipofectamine 2000 (Invitrogen, Carlsbad, CA, USA) according to the manufacturer's instructions [10]. Conditioned medium containing viral particles was harvested at 48 h and 72 h after transfection and filtered through 0.45 *μ*m filters. Cells were then infected with recombinant lentivirus in the presence of 6 *μ*g/ml polybrene (Sigma-Aldrich, H9268, Shanghai, China). After infection for 48 h, cells were selected with 2 *μ*g/ml puromycin (Gibco, A1113802, USA) for 7 days to eliminate the uninfected cells and thus yield mass populations of puromycin-resistant cells expressing the shRNAs.

### 2.4. HE Staining and Immunohistochemistry

Hematoxylin and eosin (HE) staining was done in a routine method with hematoxylin stain, eosin stain, and acid alcohol differentiation solution. Immunohistochemical (IHC) analysis was performed as the following steps. Tissues were fixed in 4% paraformaldehyde and embedded in paraffin. Antigen retrieval was performed after deparaffinization by heating the slides at 100°C for 15 min in 10 mM citrate buffer (pH 6.0). Slides were incubated with appropriate primary antibody, including ERO1L (1 : 100, Abcam, ab177156), Vimentin (Servicebio, GB111308), ki67 (Servicebio, GB13030-2), and E-cadherin (Servicebio, GB13083), following hematoxylin counterstaining, whose immunoreactivity could be observed with 3,3′ diaminobenzidine tetrahydrochloride (DAB). Images were obtained using the Zeiss Axioplan 2 Fluorescence microscope.

### 2.5. Real-Time Quantitative PCR

Total RNA was isolated from pancreatic cancer cells, normal pancreatic tissues, and hepatic metastatic tissues with RNAiso Plus (Takara, Japan) and reversely transcribed through PrimeScript RT-PCR kit (Takara, Japan) according to the manufacturer's instructions. Quantitative real-time PCR was performed to determine the expression levels of ERO1L with SYBR Premix Ex Taq (Takara, Japan). PCR cycles were performed on a 7500 real-time PCR system (Applied Biosystems, Inc., USA) with the thermal cycling settings as recommendation: one initial cycle at 95°C for 2 min followed by 40 cycles of 5 sec at 95°C and 31 sec at 60°C. Gene expression was normalized to human ACTB gene transcripts. Relative mRNA expression was calculated by the 2^−ΔΔCt^ method. Primer sequences used in this study are shown as follows: ERO1L forward, 5′-GGCTGGGGA TTCTTGTTTGG-3′; ERO1L reverse 5′-AGTAACCACT AACCTGGCAGA-3′; *β*-actin forward, 5′-ACTCGTCA TACTCCTGCT-3′, *β*-actin reverse, 5′-GAAACTACCT TCAACTCC-3′.

### 2.6. Migration and Invasion Assays

Migration and invasion assays were performed with PDAC cells, through a 6.5 mm chamber with 8 *μ*m pores (Corning, Corning, NY, USA). For migration assays, cells were added into upper chambers with noncoated membranes. For invasion assays, cells were placed on top chamber inserts precoated with 100 *μ*l 2% Matrigel (BD Biosciences, Franklin Lakes, NJ, USA). A total of 5 × 10^4^ cells suspended in 200 *μ*l serum-free DMEM were added into upper chambers for both assays, with 500 *μ*l DMEM with 10% FBS added to the lower chamber. After 24 h at 37°C, cells that migrated into or invaded the underside of membranes were fixed with 4% paraformaldehyde, stained with 0.5% crystal violet for 30 min at 37°C, washed with PBS, and counted. At least six random fields of a phase-contrast microscope (Olympus, Tokyo, Japan) were observed at ×100 magnification, and counted for each chamber. Experiments were performed three times in triplicate.

### 2.7. Western Blotting

Proteins were extracted using RIPA buffer (.5 M Tris-HCl, pH 7.4, 1.5 M NaCl, 2.5% deoxycholic acid, 10% NP-40 and 10 mM EDTA, Millipore) and separated by sodium dodecyl sulfate polyacrylamide gel electrophoresis. Anti-ERO1L (ab177156, Abcam), *β*-actin (ab8226, Abcam,) Vimentin (Servicebio, GB111308), and E-cadherin (Servicebio, GB13083) antibodies were used. *β*-Actin was used as the loading control. The next day, the membranes were incubated with species-specific secondary antibodies (ThermoFisher Scientific, USA). Finally, bound secondary antibodies were detected by Odyssey imaging system (LI-COR Biosciences, Lincoln, NE, USA).

### 2.8. Animal Experiments

Balb/c nude mice aged 6 weeks were applied for PDAC hepatic metastatic xenograft experiment. In intrasplenic inoculation model, 2 × 10^6^ cells (Panc02 cells) resuspended in 25 *μ*l PBS were injected into the spleen of Balb/c nude mice (seven mice per group). After three weeks, mice from each group were humanely sacrificed for PDAC hepatic metastatic tumor evaluation and histopathological studies. The livers were dissected, fixed with 4% paraformaldehyde, embedded in paraffin, and subjected to HE staining. Mice were manipulated and housed according to protocols approved by the East China Normal University Animal Care Commission. All mice received humane care according to the criteria outlined in the Guide for the Care and Use of Laboratory Animals prepared by the National Academy of Sciences and published by the National Institutes of Health.

### 2.9. Statistics

All data are presented as mean ± standard deviation for at least 3 repeated individual experiments for each group using SPSS 19.0 (Chicago, USA) or GraphPad Prism (San Diego, CA). Comparisons between 2 groups were made using Student's *t* test. The log-rank test was used to compare Kaplan-Meier curves. Gene-set enrichment analysis (GSEA) was carried out using the GSEA software (http://software.broadinstitute.org/gsea/index.jsp). A *P* value less than 0.05 were considered statistically significant.

## 3. Results

### 3.1. ERO1L Is Overexpressed in Metastatic PDAC and Related to DFS

**Figure 1:**
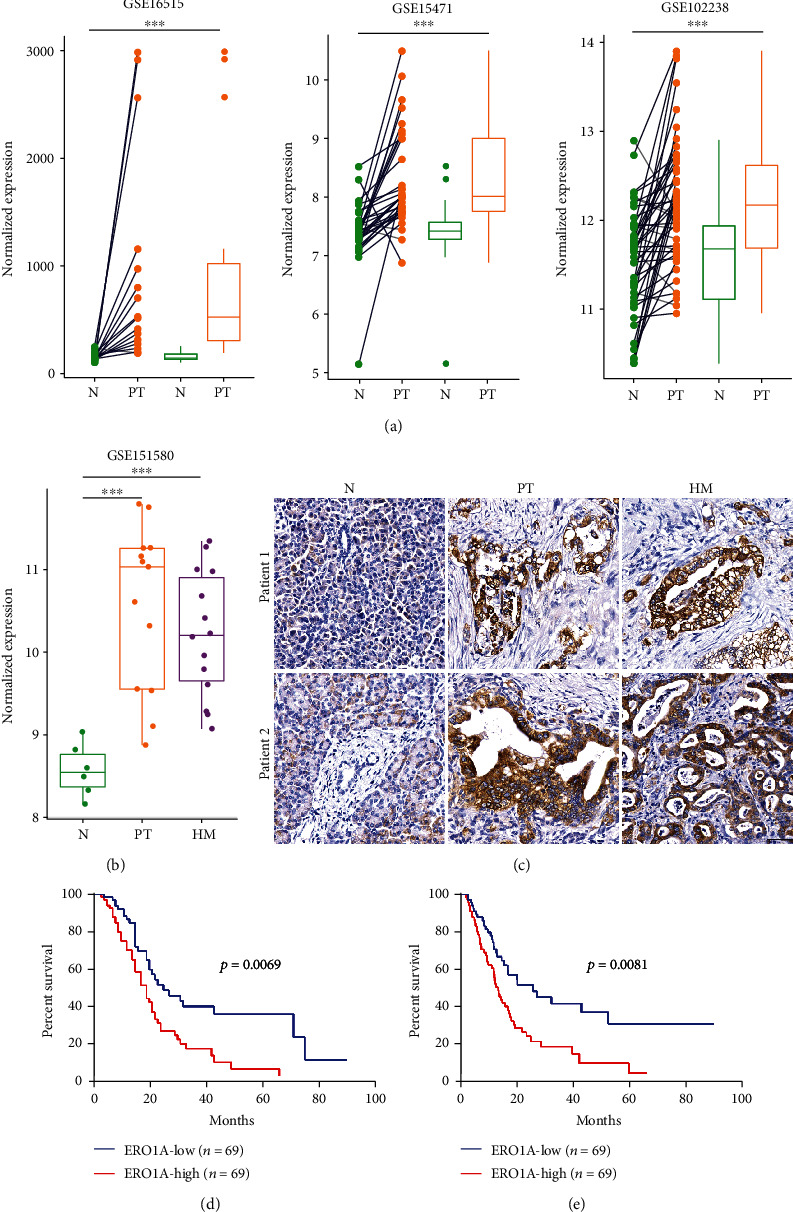
ERO1L is overexpressed in metastatic PDAC and predicts a poor prognosis. (a) The expression pattern of ERO1L in paired samples of three independent PDAC cohorts using GSE16515 (*n* = 16, *P* = 0.002752), GSE15471 (*n* = 33, *P* = 4.513*e* − 06), and GSE102238 (*n* = 47, *P* = 6.582*e* − 07). Data were derived from the GEO database. (b) The expression pattern of ERO1L in hepatic metastatic PDAC cohort using GSE151580 (*n* = 47, *P* = 2.309*e* − 06). Data were derived from the GEO database. (c) Representative images of ERO1L protein expression in two hepatic metastatic PDAC patients; Scale bar: 50 *μ*m. (d) Kaplan-Meier survival curves showing the overall survival of PDAC patients based on ERO1L expression in TCGA-PAAD datasets (log-rank test, *P* = 0.0069). (e) Kaplan-Meier survival curves showing the disease-free survival of PDAC patients based on ERO1L expression in TCGA-PAAD datasets (log-rank test, *P* = 0.0081).

To illustrate the expression pattern of ERO1L in PDAC, we firstly searched the mRNA expression level of ERO1L in three GEO datasets. The results showed that ERO1L expression was significantly upregulated in PDAC tissues comparing with paired normal pancreatic tissues using GSE16515 (Figure 1(a), *n* = 16, *P* = 0.002752), GSE15471 (Figure 1(a), *n* = 33, *P* = 4.513*e* − 06), and GSE102238 (Figure 1(a), *n* = 47, *P* = 6.582*e* − 07). Further analysis showed the expression of ERO1L was also remarkably higher in the hepatic PDAC tissues than the normal pancreas as revealed by GSE151580 (Figure 1(b), *n* = 47, *P* = 2.309*e* − 06). To further determine the protein expression of ERO1L in PDAC and hepatic metastatic PDAC cohorts, we performed IHC analysis in two hepatic PDAC patients. As a result, ERO1L was highly expressed in primary and hepatic metastatic PDAC tissues compared to adjacent nontumor tissues (Figure 1(c)). To evaluate the prognostic significance of ERO1L in PDAC patients, the correlation between ERO1L expression and corresponding clinical follow-up information were analyzed by Kaplan-Meier analysis and log-rank test in TCGA-PAAD datasets. As shown in Figures 1(d) and 1(e), patients with higher ERO1L level had significantly shorter overall survival (OS) (log-rank test, *P* = 0.0069) and disease-free survival (DFS) (log-rank test, *P* = 0.0081) time. In brief, these data showed that high expression of ERO1L might be related to distant metastasis of PDAC.

### 3.2. Genetic Silencing of ERO1L Suppresses Cell Migration and Invasion of PDAC In Vitro

**Figure 2:**
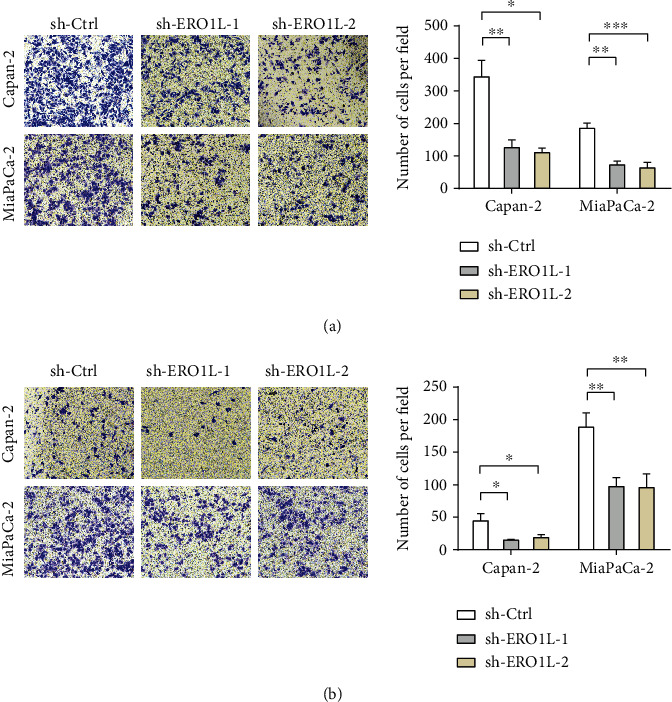
Genetic silencing inhibition of ERO1L suppresses migration and invasion of PDAC in vitro. (a) Transwell assays without coated Matrigel showing the role of ERO1L knockdown in PDAC cell migration in vitro. (b) Transwell assays with coated Matrigel showing the role of ERO1L knockdown in PDAC cell invasion in vitro.

To confirm the role of ERO1L in pancreatic cancer migration and invasion, we downregulated ERO1L in the indicated pancreatic cells using shRNAs [10]. Two PDAC cell lines with relatively higher ERO1L expression, Capan-2 and MiaPaca-2 cells, were selected for loss-of-function study. Stable expression of two short hairpin RNA (sh-1, sh-2) targeting ERO1L resulted in >80% decrease in ERO1L expression of Capan-2 and MiaPaca-2 cells [10]. The transwell assay without coated Matrigel suggested that ERO1L knockdown significantly inhibited pancreatic cancer cell migration (Figure 2(a)), whereas the transwell assay with coated Matrigel suggested that ERO1L knockdown significantly suppressed pancreatic cancer cell invasion (Figure 2(b)).

### 3.3. Pharmacological Inhibition of ERO1L Suppresses Cell Migration and Invasion of PDAC In Vitro

**Figure 3:**
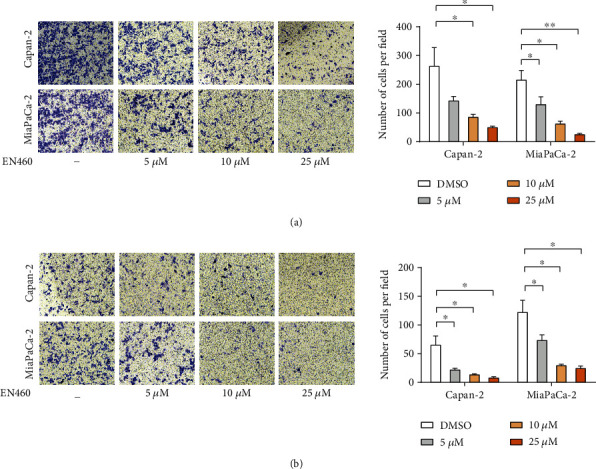
Pharmacological inhibition of ERO1L suppresses migration and invasion of PDAC in vitro. (a) Transwell assays with/without coated Matrigel showing the role of ERO1L inhibitor, EN460 in PDAC cell migration in vitro. (b) Transwell assays with/without coated Matrigel showing the role of ERO1L inhibitor, EN460 in PDAC cell invasion in vitro.

To furtherly assess the effect of ERO1L on PDAC metastasis, EN460 was used in Capan-2 and MiaPaca-2. EN460 (Billerica, MA, USA) is a known selective inhibitor of ERO1L that targets its enzymatic activity. Our data showed pharmacological inhibition of ERO1L by EN460-reduced cell viability and proliferation in a dose-dependent manner [10]. The transwell assay with/without coated Matrigel suggested that EN460 significantly inhibited pancreatic cancer cell migration (Figure 3(a)) and invasion (Figure 3(b)). The results verified that knockdown or pharmacological inhibition of ERO1L expression limits the cell migration and invasion in PDAC.

### 3.4. Pharmacological Inhibition of ERO1L Suppresses Hepatic Metastasis In Vivo

**Figure 4:**
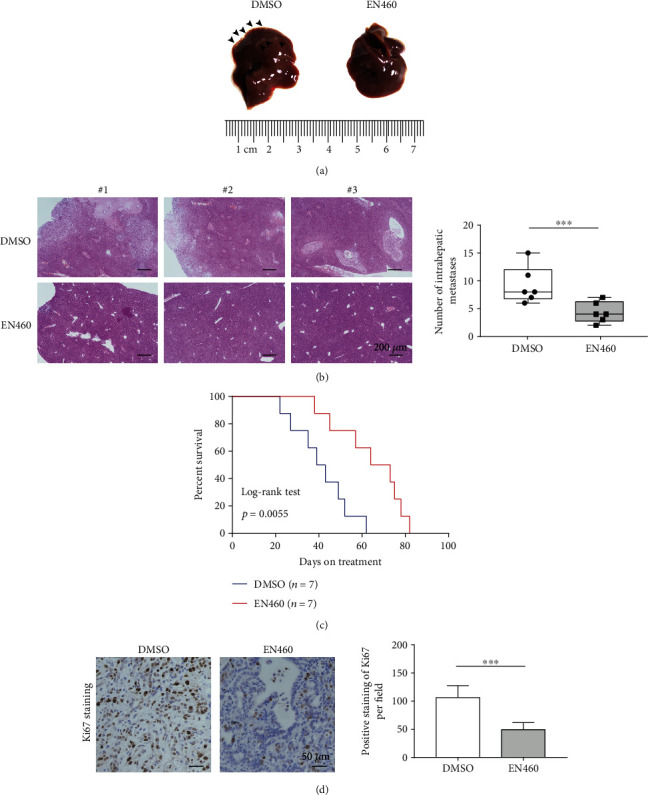
Pharmacological inhibition of ERO1L suppresses hepatic metastasis in vivo. a, b In vivo growth assay suggesting that ERO1L inhibitor, EN460 significantly decreased the number of liver nodules from Panc02 cell transplanted mice. Gross xenograft (a) and HE-staining images (b) were shown. (c) Kaplan-Meier survival curves showing the survival time of Panc02 PDAC cell transplanted mice treated with/without EN460 (log-rank test, *P* = 0.005). (d) Representative immunohistochemistry images showing EN460 suppressed liver nodules growth of PDAC in vivo. Scale bar: 50 *μ*m.

To investigate the role of ERO1L in distant metastasis in vivo, nude mice were transplanted with Panc02 PDAC cells. The number of nodules in the liver from Panc02 cell transplanted mice intraperitoneal injected with EN460, was significantly decreased, compared with the control groups (Figures 4(a) and 4(b)). In addition, EN460 also extended significantly the survival time of the mice (Figure 4(c)). Our previous study showed pharmacological inhibition of ERO1L suppresses tumor growth in PDAC in vitro. The IHC assays confirmed the weaker proliferation marker Ki67 in liver metastatic xenotumor (Figure 4(d)), which suggested EN460 also suppressed tumor growth in PDAC in vivo. Collectively, these results suggest that ERO1L might act as a prometastasis factor in PDAC.

### 3.5. EMT Contributes to ERO1L-Mediated Distant Metastasis

**Figure 5:**
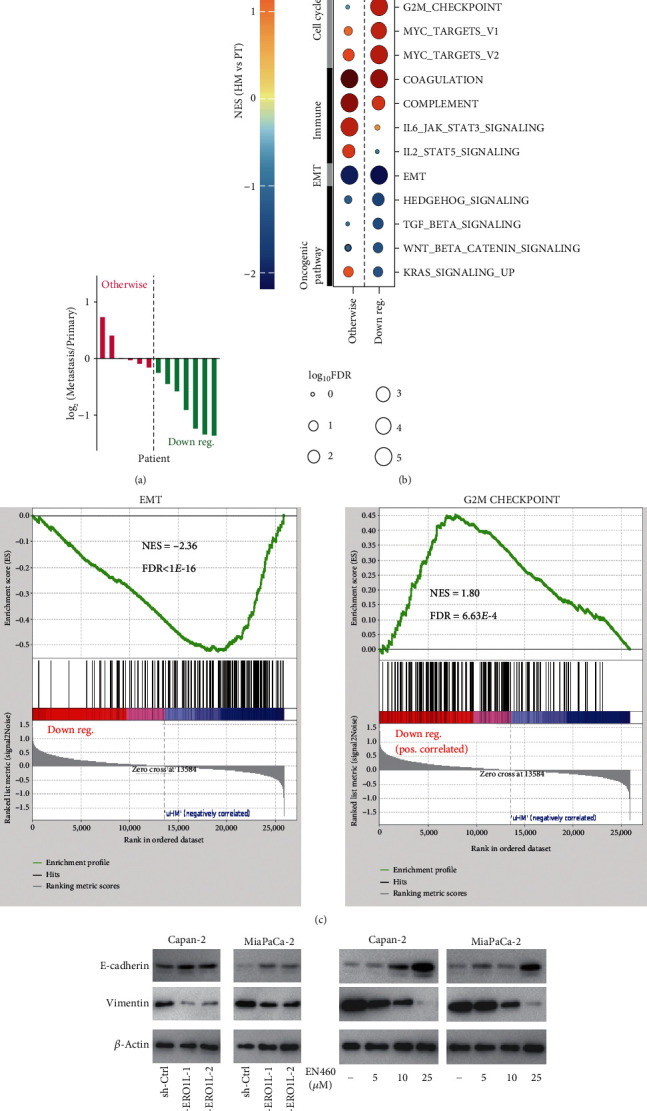
EMT contributes to ERO1L-mediated hepatic metastasis of PDAC. (a) Down reg (log2(HM/PT) < −0.25) and otherwise (log2(HM/PT) ≥ −0.25) were divided, based on the rate of ERO1L expression in hepatic metastasis and primary tumor. (b) The relatively lower ERO1L expression in hepatic metastasis leading to the lower activity of EMT. (c) GSEA suggesting lower ERO1L expression in hepatic metastasis would remarkably inhibit the activity of EMT pathway and whereas improve the activity of G2M CHECPIONT pathway positively correlated with pancreatic cancer metastasis. (d) Western blotting analysis of Capan-2 and MiaPaCa-2 cells after inhibiting ERO1L expression with shRNAs and EN460.

To examine the regulatory mechanism of ERO1L in hepatic metastasis of pancreatic cancer, we first analyzed its effect on signaling related to cancer in our RNA-sequencing data of hepatic metastatic PDAC [12]. Basing on the rate of ERO1L expression in hepatic metastasis and primary tumor, we divide patients with liver metastases into two groups: downregulation group (down reg) and otherwise group (Figure 5(a)). When log2(HM/PT) < −0.25, patients were defined as down reg. When log2(HM/PT) ≥ −0.25, patients were defined as otherwise. We observed a similar trait that the lower metastatic but higher proliferative capabilities of hepatic metastasis were compared to primary tumor, which was independent of ERO1L expression. But the relatively lower ERO1L expression in hepatic metastasis also caused unique pathway changes. It is worth noting that the activity of EMT was remarkably lower in down reg (Figure 5(b)), which was also demonstrated by GSEA (Figure 5(c)). And in addition, we discovered lower ERO1L expression in hepatic metastasis would remarkably improve the activity of G2M CHECPIONT pathway (Figure 5(c)). Mechanistically, shRNAs and EN460 were used to inhibit the ERO1L expression in Capan-2 and MiaPaca-2; the result showed the EMT-related protein Vimentin and E-cadherin were significantly changed (Figure 5(d)). The result suggested ERO1L act as an important promoting role in invasion, dissemination, colonization, and growth of hepatic metastasis in PDAC.

## 4. Discussion

In this study, we mainly explored the role of ERO1L in hepatic metastasis of PDAC. Firstly, we found that ERO1L was significantly upregulated in PDAC hepatic metastatic and primary tumor tissues compared with adjacent nontumor tissues. TCGA-PAAD dataset analysis indicated that high expression of ERO1L might be related with distant metastasis of PDAC and poor prognosis. Further study demonstrated that ERO1L made contribution to the migration and invasion of PDAC in vitro and in vivo. Mechanistically, sequencing data of hepatic metastatic PDAC analysis and experimental verification indicated ERO1L promoted invasion, dissemination, colonization, and growth of hepatic metastasis of PDAC through EMT. Making a comprehensive view of all these findings, it is not difficult to find ERO1L might be a potential target of PDAC hepatic metastasis.

PDAC is characterized by intense hypoxia and high unfolded protein response (UPR) activation status [13]. Exposure to diverse ER stress, cancer cells have to maintain the ER homeostasis via the dynamic intracellular UPR signaling pathway [11]. Among the oxidoreductases expressed in the ER, ERO1L is central to oxidative protein folding, but its role in tumor progression was unclear [14]. In recent years, emerging evidence has verified ERO1L acted as an important role for tumor. In breast cancer cases, ERO1L also proved to be able to promote angiogenesis by augmentation of VEGF production [7], support tumoral immune escape through PD-1 [15], and serve as a novel predictor for poor prognosis of breast cancer [14]. In our previous study, we revealed that ERO1L expression is dramatically upregulated by activation of UPR and could be induced by hypoxia. We had demonstrated that ERO1L plays a glycolysis-dependent growth-promoting role in PDAC. In the current study, we furtherly analyzed TCGA data and GEO database to confirm that highly expressed ERO1L is correlated with a poor prognosis in PDAC patients. The evidence proved the promoting role of ERO1L in tumor, especially PDAC.

Metastasis is the most important trait of PDAC and a complex multistep process from local invasion to finally colonization and outgrowth in distant body site [16]. ERO1L serves as an oncogenic-promoting effector and play versatile oncogenic functions in human malignancies [5, 10]. But its role and exact mechanism on tumor metastasis remain unclear. In HCC, it has proved that ERO1L can promote the migration, invasion, and lung metastasis of HCC in vitro and in vivo through the S1PR1/STAT3/VEGF-A pathway [11]. Han et al. preliminarily suggested the promoting metastasis role of ERO1L in PDAC [17]. TCGA-PAAD dataset analysis indicated that high expression of ERO1L might be related with DFS. ERO1L was significantly upregulated in PDAC hepatic metastatic tissues. These positive clues suggested the promotion of metastasis role of ERO1L in PDAC. We further clarified the promoting metastatic effect of ERO1L in PDAC in vitro and in vivo. Furthermore, pharmacological inhibition with EN460 also could reduce the invasion, migration, and liver metastasis of PDAC. Bioinformatics analysis of large-scale metastatic PDAC sequencing showed ERO1L expression could regulate many signaling pathways such as Wnt/*β*-catenin, G2M CHECKPOINT, E2F TARGETS, MYC TARGETS, and EMT. EMT played crucial role in tumor metastasis [18–21]. We confirmed that the knockdown or pharmacological inhibition of ERO1L could inhibit the invasion, migration, and liver metastasis of PDAC through EMT. In addition, lower ERO1L expression in hepatic metastasis compared to primary tumor would remarkably improve the activity of G2M CHECPIONT pathway. This result indicated the change of ERO1L expression might supply a growth advantage for dissemination PDAC cells and further demonstrated the promoting metastasis role of ERO1L in multistep metastatic process.

In conclusion, we have revealed the expression pattern and prognostic value of ERO1L in PDAC and have demonstrated a novel function of ERO1L in PDAC hepatic metastasis. Knockdown or pharmacological inhibition of ERO1L demonstrated ERO1L could regulate liver metastasis of PDAC through EMT. Our study on EMT suggested the pivotal role of ERO1L in progression and metastasis of PDAC. Thus, strategies designed to treat ERO1L as a novel prognostic indicator and potential therapeutic target for PDAC may be promising.

## Data Availability

The data used to support the findings of this study are included within the article.
